# Epigenetic downregulation of the ISG15–conjugating enzyme UbcH8 impairs lipolysis and correlates with poor prognosis in nasopharyngeal carcinoma

**DOI:** 10.18632/oncotarget.6218

**Published:** 2015-10-12

**Authors:** Xiaoying Zhou, Jiazhang Wei, Fu Chen, Xue Xiao, Tingting Huang, Qian He, Shumin Wang, Chunping Du, Yingxi Mo, Longde Lin, Ying Xie, Lili Wei, Ying Lan, Mairiko Murata, Guangwu Huang, Ingemar Ernberg, Liudmila Matskova, Zhe Zhang

**Affiliations:** ^1^ Department of Otolaryngology-Head & Neck Surgery, First Affiliated Hospital of Guangxi Medical University, Guangxi, China; ^2^ Department of Microbiology, Tumor and Cell Biology, Karolinska Institutet, Solna, Sweden; ^3^ Department of Radiation Oncology, Eye Ear Nose & Throat Hospital of Fudan University, Shanghai, China; ^4^ Department of Environmental and Molecular Medicine, Mie University Graduate School of Medicine, Mie, Japan; ^5^ School of Public Health, Guangxi Medical University, Guangxi, China

**Keywords:** UBE2L6, nasopharyngeal carcinoma, DNA methylation, tumor suppressor gene, ATGL

## Abstract

We identified the *UBE2L6* gene, encoding the ISG15-conjugating enzyme UbcH8, as one gene significantly downregulated by promoter hypermethylation in nasopharyngeal carcinoma (NPC). Reduced expression of the UbcH8 protein correlated with poor outcome in NPC patients. Restored expression of *UBE2L6* suppressed proliferation and colony formation in NPC cells, while inducing apoptosis. Of particular interest, we found that aberrant lipid turnover was controlled by UbcH8 in NPC through ISG15-conjugation of valosin-containing protein (VCP). Tumor tissue and NPC cell lines showed conspicuously strong accumulation of lipid droplets (LDs) compared to control nasopharyngeal epithelium and non-cancerous cell lines. We demonstrated that UbcH8 counteracts degradation of adipocyte triglyceride lipase (ATGL), a key enzyme in lipid catabolism.

## INTRODUCTION

Nasopharyngeal carcinoma (NPC) is a unique head and neck cancer with a distinctive ethnic and geographic distribution and a complex etiology. During development and progression of NPC, latent Epstein-Barr virus (EBV) infection, multiple genetic and epigenetic changes synergistically perturb normal cellular functions, thus contributing to NPC pathogenesis [1].

In human cancers, the coincidence of genome-wide hypomethylation with gene-specific hypermethylation in CpG islands of gene promoter regions has been well documented [2]. To date, a series of putative tumor suppressor genes (TSGs) inactivated by promoter hypermethylation have been discovered. These tumor suppressors are involved in many cellular pathways implicated in NPC, including cell cycle regulation [3], apoptosis [4], cell adhesion [5, 6], and signal transduction [7, 8].

In the present study, we identified 479 genes, which were epigenetically down-regulated in NPC cell lines. We focused our interest at the *UBE2L6* gene, which showed a strong down-regulation. This gene encodes an E2 enzyme (UbcH8) which participates in both ubiquitylation and ISG15ylation [9–11].

Interferon-stimulated gene 15 (ISG15) has been identified to be a potential serological marker for cancer [12]. Conjugated ISG15 has been suggested to promote tumorigenesis, whereas free ISG15 is tumor suppressive [13–15]. This emphasizes the importance of exploring ISG15 modifying enzymes. Thus far, only two E2 enzymes, UbcH6 and UbcH8 have been confirmed to conjugate ISG15 [16–18]. Only a few UbcH8 ISG15-targets have been identified so far [19].

Interestingly, knock-down of *UBE2L6* in mice demonstrated a major impact on lipid metabolism, which repressed the differentiation program of adipocytes [20]. Impairment of Adipose triglyceride lipase (ATGL) expression and/or function, as the crucial enzyme initiating lipid digestion, results in lipid droplets (LDs) accumulation [21]. A functional link between *UBE2L6* and ATGL has been established although the molecular mechanism of the link is not yet fully elucidated. Investigation of ATGL protein turnover pointed to the ATPase valosin-containing protein (VCP) as a necessary factor in ATGL unfolding for the sequential degradation by proteasomes [22]. VCP has also been shown to be a target of ISG15 in a large-scale screen for ISG15 modified proteins [23]. Inspired by the fact that *UBE2L6* can act as an ISG15-conjugating enzyme, and by the dysfunction of lipid turnover in *UBE2L6* knock-out mice [20], we postulated that *UBE2L6* could control ATGL stability through ISG15 ligation to VCP.

LDs is a dynamic organelle recently observed to be abnormally accumulated in human tumor tissues [24]. Accumulation of LDs in the cytoplasm is a result of impaired metabolism in tumor cells [25]. Although the regulation and function of LDs in non-adipocytes is unclear, it is obvious that lipids supply fuel energy in cancer cells. Lipid mediators derived from tumor cells play a critical role in inducing chronic inflammation in the tumor microenvironment [26].

We have now shown that *UBE2L6* is frequently down-regulated in NPC derived cell lines and primary tumors by promoter hypermethylation. Reduced expression of the UbcH8 protein correlated with poor prognosis in NPC patients. *UBE2L6* was verified as a candidate TSG as it significantly suppressed proliferation, colony formation and induced apoptosis in NPC cells. This phenotype could be related to the fact that UbcH8 stabilizes ATGL through ISG15ylation of VCP, since this modification has an inhibitory effect on VCP activity. In summary, we show that epigenetic silencing of UbcH8 may play an interesting role in NPC carcinogenesis by affecting lipid metabolism.

## RESULTS

### *UBE2L6* is inactivated in NPC cell lines and primary tumors

**Figure 1:**
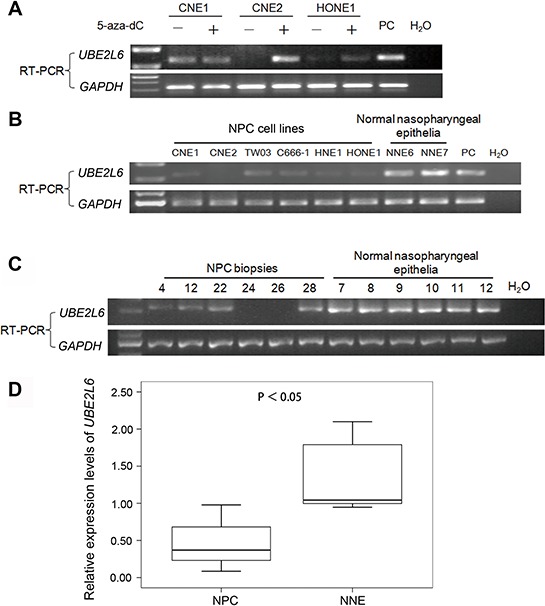
Transcriptional level of UBE2L6 in NPC and NNE **A.** Treatment with 5-aza-dC alone restores the expression of *UBE2L6* in NPC cell lines. **B.** Expression levels of *UBE2L6* in NPC cell lines and normal nasopharyngeal epithelia (NNE) were analyzed by RT-PCR. **C.** Semi-quantitative RT-PCR analysis of *UBE2L6* mRNA expression in primary NPC tumors and NNEs. Representative examples are six primary NPC tumors and six NNEs. PC: Specific UBE2L6 expression plasmid DNA was used as a positive control. **D.** Summary of *UBE2L6* expression in primary NPC (*n* = 37) tumors and NNEs (*n* = 12). The box plots show the ratios of the intensities of *UBE2L6* and *GAPDH* signals. Boxes indicate 25 to 75 percentile, horizontal line indicates the mean, and bars indicate 10 and 90 percentile.

cDNA microarray was performed to screen for down-regulated genes, possibly inactivated by promoter hypermthylation in two NPC cell lines CNE2 and HONE1 (Fig. 1). Among the candidate genes identified, the transcription of *UBE2L6* showed a strong increase (up to 7.8-fold) after 5-aza-dC and TSA treatment in both cell lines. We performed semi-quantitative reverse transcription-PCR (RT-PCR) to validate the microarray data on three NPC cell lines (CNE1, CNE2 and HONE1) (Fig. 1A).

Further, we tested *UBE2L6* transcription levels in six cell lines (CNE1, CNE2, TW03, HNE1, HONE1 and C666-1), 37 NPC primary tumor biopsies and 12 normal nasopharyngeal epithelium by RT-PCR. All of the normal nasopharyngeal epithelia expressed an easily detectable level of *UBE2L6* mRNA. Among the NPC cell lines, *UBE2L6* expression was undetectable in CNE2, while the other five had weak expression of *UBE2L6* (Fig. 1B). *UBE2L6* mRNA was completely silenced in 5 of the 37 primary NPC tumor biopsies. The overall expression levels of *UBE2L6* was significantly lower in the 37 NPC tumor biopsies as compared to the 12 non-malignant nasopharyngeal epithelium (NNE) tested (*p* < 0.05, Fig. 1C, 1D).

### UbcH8 expression is downregulated in NPC and correlates with patient survival

**Figure 2:**
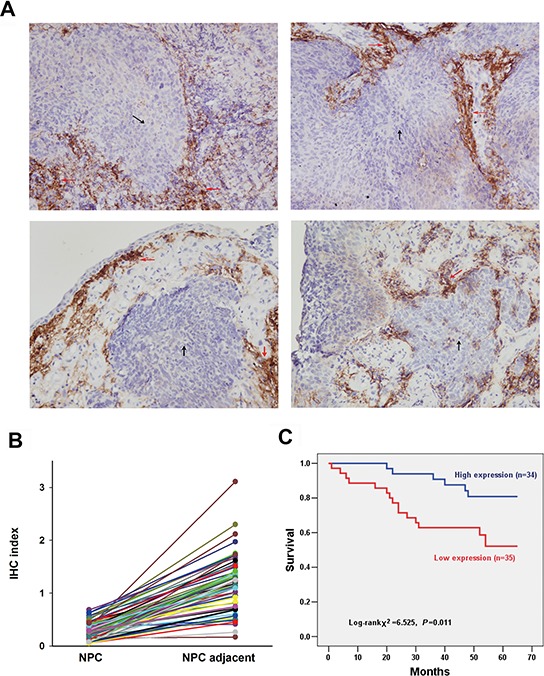
Detection of UbcH8 expression in NPC biopsies by immunohistochemistry **A.** Representative slides of NPC samples stained by anti-UbcH8 antibody. Black arrows denote NPC tissue and red arrows denote adjacent stromal tissue. IHC indexes of NPC vs. NPC adjacent: upper left (0.39 vs. 1.18); upper right (0.18 vs. 0.51); lower left (0.53 vs. 1.75); lower right (0.57 vs. 1.40). **B.** IHC indexes of all the investigated NPC biopsies with paired cancer nest and corresponding adjacent non-cancerous stromal tissue (*n* = 69). **C.** Kaplan-Meier survival curves illustrating the significance of UbcH8 expression in NPC. NPC patients with low UbcH8 expression had shorter NPC-specific survival than those with high UbcH8 expression (*p* = 0.011).

UbcH8 expression was studied in a total of 69 NPC tumor tissues. Based on immunohisto-chemical analysis, positive staining for UbcH8 was predominantly observed in the cytoplasm and to a lesser degree in the nuclei of non-cancerous stromal cells. UbcH8 expression levels in NPC cancer nests were significantly lower than in adjacent stromal tissue (Fig. 2A, 2B).

At the end of follow-up, 21 of 69 patients (30.4%) died from NPC, 45 of 69 patients (65.2%) remained alive, and 3 of 69 patients (4.4%) died from other causes or could not be contacted. Among the 69 NPC specimens, 35 (50.7%) specimens were defined with low expression, and 34 (49.3%) specimens with high expression. Univariate Kaplan-Meier survival analysis of the complete NPC patients (*n* = 69) based on UbcH8 expression indicated that the disease-specific survival period was significantly shorter for patients with low UbcH8 expression than for patients with high UbcH8 expression (*p* = 0.011; Fig. 2C).

### The *UBE2L6* promoter is hypermethylated in NPC cell lines and primary tumors, but not in normal epithelia

**Figure 3:**
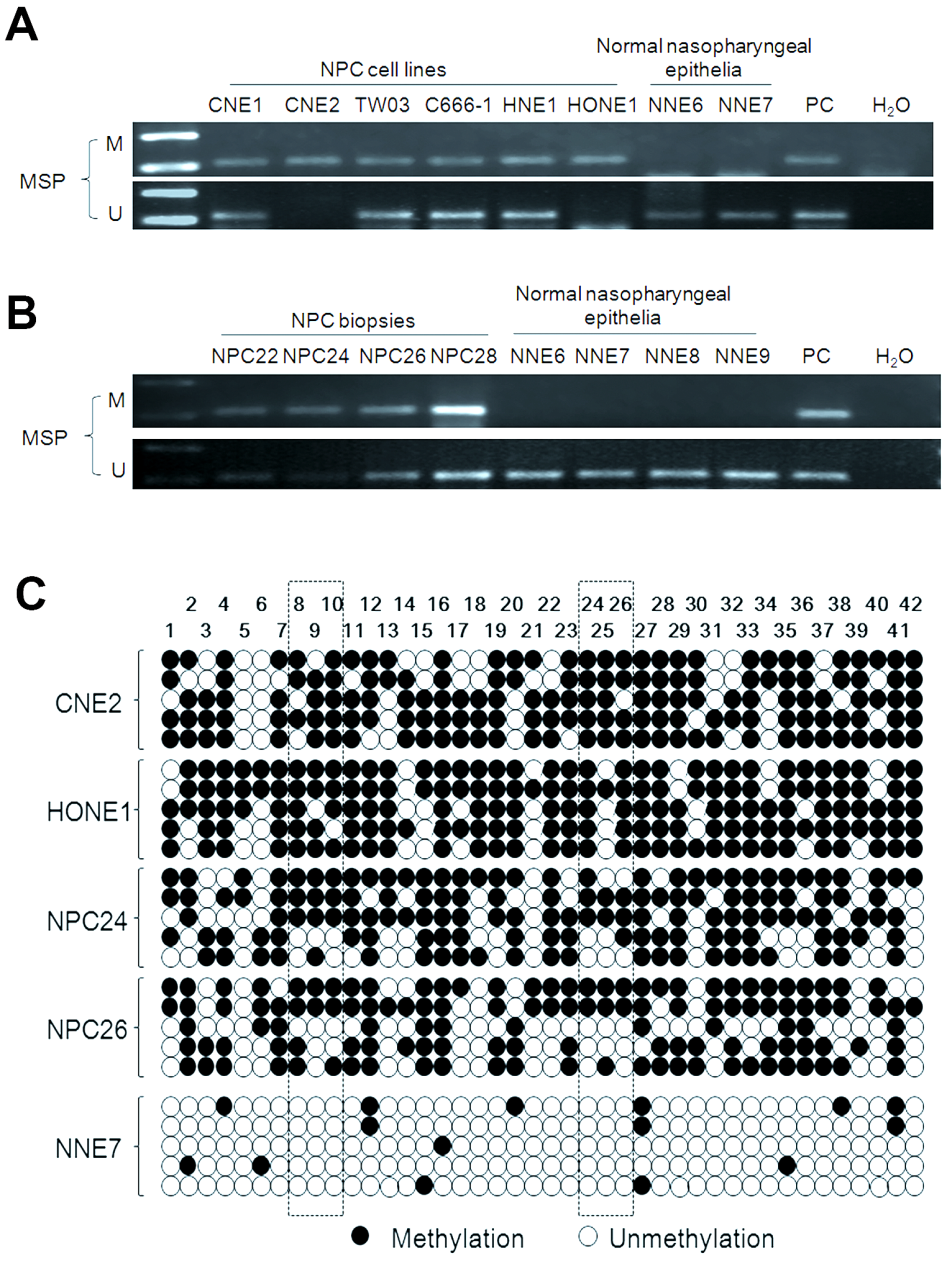
Methylation-specific PCR analysis of the UBE2L6 promoter region in NPC cell lines, NPC primary tumors and normal nasopharyngeal epithelium U: unmethylated alleles; M: methylated alleles. PC: positive control. *In vitro* methylated DNA was used as methylation positive control and DNA from normal lymphocytes was used as a positive control for unmethylated alleles. **A.** Methylation status of the *UBE2L6* promoter region in NPC cell lines and NNE. **B.** Methylation status of the *UBE2L6* promoter region in NPC primary tumors and NNE. 4 NPC primary tumors (NPC 22, 24, 26 and 28) and 4 NNE (NNE6, 7, 8 and 9) are shown as examples. **C.** Methylation status of the 42 CpG sites within the *UBE2L6* gene promoter in two NPC cell lines (CNE2 and HONE1), two NPC biopsies (NPC24 and NPC26) and one normal nasopharyngeal epithelium biopsy (NNE7) were analyzed by bisulphite genomic sequencing. Five randomly selected clones were sequenced for each sample. Each row represents an individual promoter allele analyzed. Open circles denote unmethylated cytosines, and closed circles denote methylated cytosines. The frames show the CpG pairs covered by MSP primers.

To address the mechanism of inactivation of *UBE2L6* gene in NPC, we analyzed the methylation status of the *UBE2L6* promoter in six NPC cell lines, 40 primary tumors and 12 normal nasopharyngeal epithelium by methylation specific PCR (MSP). Hypermethylation was found in all these NPC cells, in 47.5% (19/40) of the primary NPC tumor biopsies, but in none of the normal nasopharyngeal epithelia (Fig. 3A, 3B). It should be noted that unmethylated fragments were detected in some of the NPC cell lines and biopsy samples. This could be due to heterogeneity among cells and reflect the presence of stromal non-malignant cells in a fraction of the tissue samples or to allelic difference in methylation modification in the NPC cell lines.

In order to determine the methylation status of individual CpG sites in the NPC samples relative to the non-malignant control biopsies, the detailed methylation status of the *UBE2L6* promoter region −204 to +156 bp, containing 42 CpG sites, relative to the translation start site, was determined by bisulphite genomic sequencing. Our results showed that most CpG sites were methylated in the NPC cell lines (CNE2 and HONE1) and the NPC biopsies (NPC24 and NPC26) (Fig. 3C). The normal nasopharyngeal epithelia (NNE7) showed single, randomly distributed methylated CpGs.

We detected no significant difference between tumors with methylated or unmethylated *UBE2L6* promoters and association with age, sex, cancer staging, lymph node metastasis or pathological subtypes (data not shown).

### *UBE2L6* suppresses cell proliferation and induces apoptosis in NPC cells

**Figure 4:**
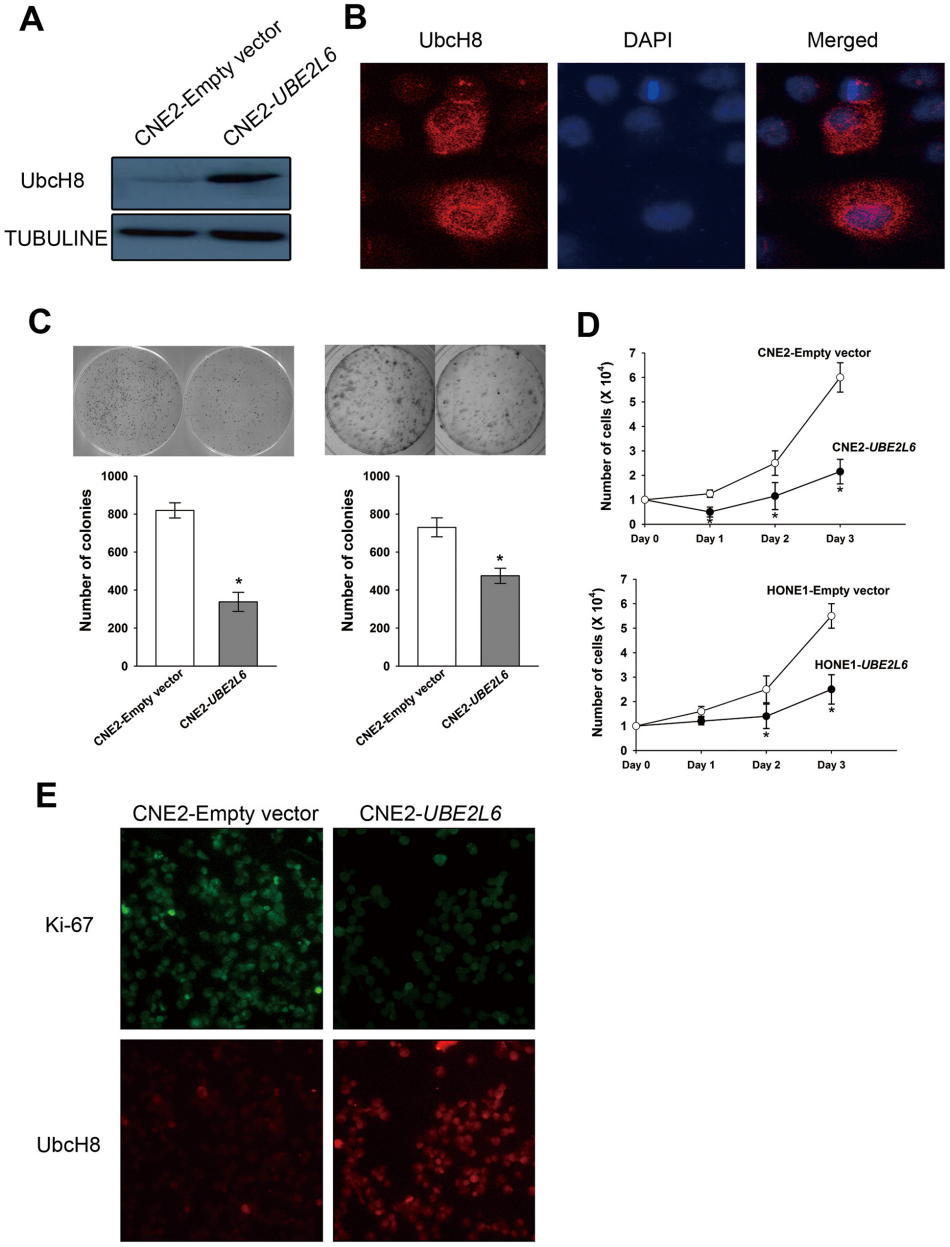
Ectopic expression of UBE2L6 in NPC cells inhibits colony formation and proliferation **A.** Western blot of transient transfectants of CNE2-*UBE2L6* and CNE2-Empty vector. **B.** Intracellular location of UbcH8 in CNE2 cells. (× 400) **C.** Colony formation assay. After selection with G418 for two weeks, colonies with stable transfected cells were form. Colony numbers shown in the bar graph represent the mean ± s.d. of three independent dishes. **D.** Growth curves of stable transfectants of CNE2 and HONE1 cells transfected with *UBE2L6* and empty vector. **E.** Immunofluorescence staining of UbcH8 and Ki-67 antibody in transient transfected CNE2-*UBE2L6* and CNE2-Empty vector cells. (× 200). *: *p* < 0.05.

The fact that *UBE2L6* is frequently subject to epigenetic silencing in NPC indicates that *UBE2L6* may function as a TSG in NPC. To substantiate this we analyzed the gene expression profile in *UBE2L6* transiently transformed CNE2 cells, confirmed by Western blot and immunofluorescence staining (Fig. 4A, 4B, Figs. 2). We detected a significant impact of *UBE2L6* on antiproliferative and proapoptotic pathway related genes. As shown in Fig. 4C, the number of colonies formed by CNE2-*UBE2L6*/HONE1-*UBE2L6* cells was less than that of CNE2-empty vector/HONE1-empty vector cells (*p* < 0.05). A cell proliferation assay was also performed, showing that the CNE2-*UBE2L6*/HONE1-*UBE2L6* cells grew significantly slower compared to CNE2 and HONE1-empty vector cells (Fig. 4D). In addition, Ki-67 immuno-staining showed a significantly decreased fluorescence-intensity in *UBE2L6*-transfected cells compared with vector-transfected cells (Fig. 4E), confirming the antagonistic effect of *UBE2L6* gene on cell proliferation in CNE2 cells.

**Figure 5:**
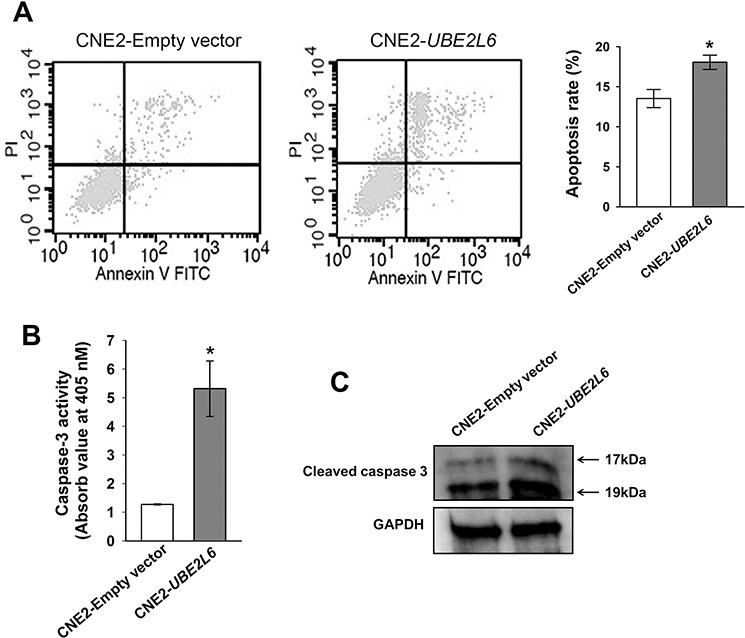
UBE2L6 induces apoptosis in NPC cells **A.** CNE2 cells were transfected with empty vector or *UBE2L6* expression vector for 24 hrs. Apoptosis were then assessed by flow cytometry after double staining with Annexin V-FITC and PI. Cells that were positively stained by Annexin V-FITC only (early apoptosis) and positive for both Annexin V-FITC and PI (late apoptosis) were quantitated, and both subpopulations were considered as apoptotic cells (mean ± s.d, *n* = 3). **B.** At 72 hrs after transfection in CNE2 cells with either empty vector or *UBE2L6*, the Caspase-3 enzyme activity was measured with Ac-DEVD-pNA by a colorimetric assay ((mean ± s.d, *n* = 3). **C.** Cleaved Caspase-3 protein was detected by western blot in transfected CNE2 cells. The arrows point to cleaved Caspase-3 fragments with molecular weights of 17kDa and 19kDa. *: *p* < 0.05.

By flow cytometric analysis, a remarkable increase of apoptosis was observed in *UBE2L6* transfected cells as compared to empty vector control. The apoptosis rate in CNE2-*UBE2L6* cells (18.06 ± 0.90%) was significantly higher than that in the empty vector control (13.52 ± 1.14%) in 3 independent tests (Fig. 5A). Caspase 3 activity assay also showed a higher apoptotic activity in *UBE2L6*-transfected cells compared with empty vector transfected cells (Fig. 5B). This data was further confirmed by Western-blotting, which demonstrated increased expression of the cleaved (activated) forms of Caspase 3 in *UBE2L6* transfected cells (Fig. 5C).

### Inactivation of *UBE2L6* impairs lipolysis by impeding ISG15ylation of VCP in NPC cells

**Figure 6:**
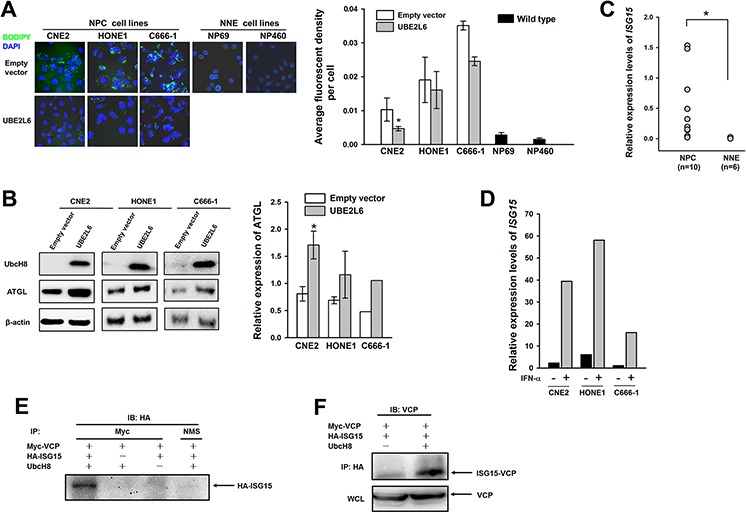
UbcH8 controls ATGL expression through ISG15ylated VCP in NPC cells **A.** Fluorescence staining with Bodipy and quantitative analysis of lipid content in NPC cells (CNE2, HONE1 and C666-1) transfected with either empty vector or *UBE2L6* plasmid. Bar graph shows a statistical evaluation of Bodipy fluorescence signal in NPC cell lines. **B.** Western blot analysis of ATGL expression in the same NPC cell lines as in A after introducing *UBE2L6* expression. Among them, C666-1 was treated with IFN-α at 500 U/mL overnight post-transfection. **C.** Relative expression of *ISG15* in biopsies by the semi quantitative qPCR. **D.** Relative expression of *ISG15* in NPC cell lines after IFN-α stimulation. **E.** Western blot analysis of VCP modification upon HA-ISG15 and Myc-UBE2L6 transient expression in CNE2 cells. NMS: normal mouse serum. **F.** Western blot analysis of HA-ISG15 and Myc-VCP protein complex in CNE2 cells. WCL: whole cell lysate. *: *p* < 0.05.

In an attempt to clarify the functional role of *UBE2L6* in NPC tumorigenesis, we stained NPC cell lines, which were characterized by impaired UbcH8 expression (Fig. S3), with a lipid specific fluorescent dye Bodipy 493/503. NPC cell lines (CNE2, HONE1 and C666-1) were stained positively, while the non-malignant cells NP69 and NP460 did not show visible LD formation. Moreover, ectopically introduced *UBE2L6* reversibly correlated with LDs. The *UBE2L6* expressing cells had significantly less LDs (Fig. 6A). LDs in cell lines were quantitatively evaluated as an average fluorescence intensity signal for single cells. ATGL expression analyzed by Western blot in NPC cell lines CNE2 and HONE1 correlated inversely with LDs and could be increased upon ectopic *UBE2L6* expression (Fig. 6B). To further confirm the correlation of *UBE2L6* expression with ATGL, we knocked down *UBE2L6* in a human embryonic kidney cell line 293, and found that ATGL protein was decreased (Fig. S4).

To investigate the ISG15ylation activities of UbcH8 involved in LDs control, we analyzed ISG15 expression in NPC tissues. NPC tumors did express significantly more ISG15 than normal adjacent tissues (Fig. 6C). ISG15 could be further induced in the cell lines upon IFN-α treatment (Fig. 6D). IFN-α treatment in combination with restoration of UbcH8 expression in NPC cell line C666-1 resulted in ATGL stabilization (Fig. 6B). Moreover, the exogenous expression of UbcH8 induced ISG15-conjugated species formation as shown by silver staining of precipitates with ISG15 antibody in NPC cells (Fig. S5). An ISG15ylation model system was reconstituted in CNE2 NPC cell line by transient transfection of Myc-*UBE2L6*, HA-ISG15 and Myc-VCP constructs, and analyzed by reciprocal immunoprecipitations. *UBE2L6* induced a strong formation of VCP-ISG15 complexes, as HA-ISG15 was detected in Myc-VCP (Fig. 6E) and ISG15-conjugated VCP in HA-ISG15 precipitates (Fig. 6F).

### NPC tissues contain more lipids than NNE

**Figure 7:**
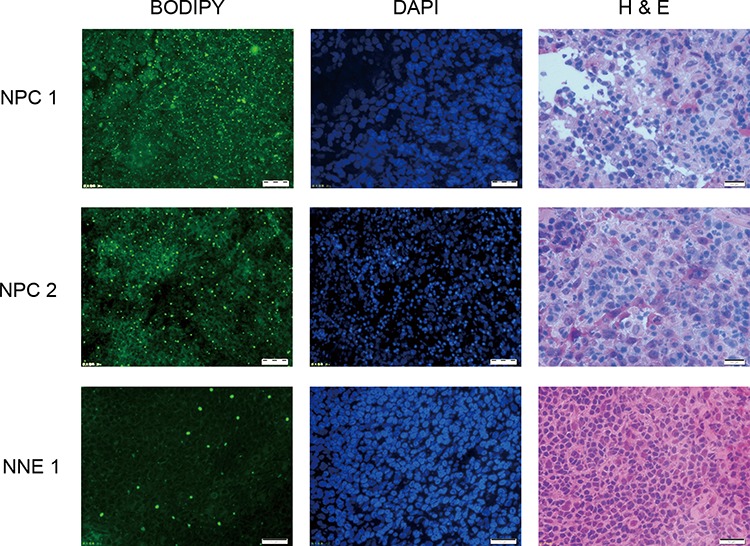
Lipid droplets (LDs) are enriched in NPC tissues but not in normal nasopharyngeal tissue Visualized by Bodipy staining (left panel) exemplified by two NPC-tissues and one control. DAPI-staining (middle panel) in the same section and HE-staining (right panel) of the next section for identification of the cells.

Here we demonstrated that LDs are accumulated *in vivo*, that NPC tumor sections were positive for LDs, as evidenced by Bodipy staining (Fig. 7).

## DISCUSSION

UbcH8 was originally discovered as a novel ubiquitin-conjugating enzyme (E2) for the HECT-domain ubiquitin ligase E6AP, which degrades p53 in HPV-infected cells [27]. Additional UbcH8 substrates have been identified by now [28, 29]. The E1 and some E3 enzymes have been identified, with which UbcH8 cooperates. It has become clear that UbcH8 shows multiple enzymatic activities [19].

UbcH8 is found to be overexpressed in esophageal squamous cell carcinoma [30]. On the other hand, depletion of the tumor suppressor gene BRCA2 results in downregulation of UbcH8 in breast cancer cells. Several studies have shown that UbcH8, induced by the histone deacetylase inhibitor (HDACi), evokes proteasomal degradation of oncogenic proteins, indicating a potential anti-tumorigenetic function in cancer [28]. In addition, we observed a significant upregulation of *UBE2L6* in HONE1 cells upon treatment with HDACi butyric acid (data not shown).

In this study, we report the first instance of epigenetic silencing of *UBE2L6* in human cancer. We demonstrate epigenetic inactivation of the *UBE2L6* gene by promoter hypermethylation in NPC cell lines and primary NPC tumors but not in normal nasopharyngeal epithelia. A corresponding reduction in UbcH8 protein expression was confirmed by immunohistochemical analysis. Moreover, *UBE2L6* expression was efficiently restored by the demethylating agent 5-aza-dC in two of three NPC cell lines tested.

Focusing on the ISG15ylating activity of UbcH8, we investigated the molecular mechanism of UbcH8 action in NPC. Based on the recent finding that the ATPase VCP is ISG15ylated [23] and that adipocyte triglyceride lipase (ATGL) degradation is controlled by VCP [28], we explored whether UbcH8 mediated ISG15ylation of VCP would affect ATGL expression. The ATPase activity of VCP is essential for segregation of polyubiquitinated misfolded or regulatory client proteins from intracellular structures for subsequent degradation by the proteasome [31]. Congruent with the findings in mouse adipocytes, we found that ectopic *UBE2L6* expression stabilizes ATGL level in NPC cells. Since it has been reported that ubiquitin- and ISG15- modification can be mutually exclusive [32], one could speculate that ISG15ylation of VCP inhibits VCP-mediated proteasomal degradation of ubiquitinated proteins. Our results are further corroborated by lipid accumulation in cells with reduced *UBE2L6* expression, providing a background to the importance of the control of ATGL protein expression in cellular homeostasis [21]. Moreover, our results provide a rational explanation for the previously reported ISG15ylation of VCP. It would be interesting to find out whether a E3 ligase of the homologous to the E6P carboxyl terminus (HECT) family, HERC5, which catalyses ISG15ylation of a panel of targets, cooperates with UbcH8 in ISG15ylation in NPC cells, as E6AP, an E3 ubiquitin ligase of HECT family, cooperates with UbcH8 in ubiquitylation [19]. This modification may either counteract binding of ubiquitinated species to VCP or induce cooperation with deubiquitinating enzymes (DUBs). Both mechanisms protect proteins from degradation. Alternatively, ISG15ylation may interfere with ATPase activity of VCP as it has been reported in other systems [33].

Interestingly, we also observed promoter methylation and silencing of another lipid binding protein, perilipin 2 (ADFP/ADRP) in NPC cells (unpublished data). ADFP is reported to mediate lipid secretion [34]. Lipid secretion may alert the immune system to suppress tumor proliferation or directly inhibit tumor growth [35]. As both UbcH8 and ADFP are differentially expressed in NPC, it is tempting to speculate that lipid homeostasis is altered at the systemic level in NPC. Enhanced de novo lipid synthesis is widely accepted by now as an early and common event in many types of cancer [36, 37]. A recent finding show that the Epstein-Barr virus-encoded small RNAs (EBERs) upregulate the cellular lipid metabolic process, contributing to the proliferation of EBV positive NPC cells [38]. In human breast and prostate cancer, overexpression of genes involved in lipid metabolism and cholesterol biosynthesis are associated with aggressive phenotype and clinical behavior [39]. Even it has been noted that lipid metabolism alteration results in tumor adaptation to antiangiogenic treatment [40]. While previous publications focus on tumor associated lipid synthesis, our data reveal impairment in lipid degradation.

The functional assays described above provide evidence that *UBE2L6* functions as a TSG in human cancers. In our case, Kaplan-Meier analysis showed a significantly shorter survival for patients whose NPC tumors had lower UbcH8 levels, thus low level of UbcH8 protein being a marker of poor prognosis for patients with NPC.

Recently it has been pointed out that a disabled ISG15-pathway decreases the cellular sensitivity to chemotherapeutic agents camptothecin (CPT), as shown in breast cancer [41]. This might extend to other chemotherapeutic regimens. Epigenetic silencing by promoter hypermethylation of TSGs can be reversed by demethylating agents. The therapeutic potential of such agents is currently the focus of several preclinical and clinical investigations [42]. In this context, UbcH8 may be a valuable therapeutic target in cancer treatment, since reactivation of UbcH8 by demethylating agents may engage both the tumor suppressive and chemosensitising activities of the UbcH8 protein. Additional studies are needed to explore this potential of *UBE2L6* as a therapeutic target for cancer treatment.

In summary, our present findings show that *UBE2L6* is inactivated by promoter hypermethylation in NPC. This causes lipid accumulation in the NPC cells due to inappropriate ATGL protein turnover. Our data indicate for the first time, that VCP ISG15ylation opposes degradation promoting function of VCP. We show that reduced expression of UbcH8 correlated with poor outcome in patients with NPC. This and other studies suggest that *UBE2L6* may be a TSG in NPC due to its ISG15-conjugating function.

## MATERIALS AND METHODS

### Ethics statement

Ethical permission of this study was approved by the Research Ethics Committee of the First Affiliated Hospital of Guangxi Medical University (Nanning, China) and by the Regional Ethics Committee of Karolinska Institutet, Stockholm, Sweden, No. 00–302.

### NPC cell lines, primary tumors and NNE

The six NPC cell lines (CNE1, CNE2, TW03, C666-1, HNE1 and HONE1) [43–46] were routinely maintained in IMDM medium (Invitrogen, Carlsbad, CA., USA) supplemented with 10% fetal calf serum (HyClone, UK Ltd, Northumberland, UK) at 37°C in an atmosphere of 5% CO_2_. Two non-malignant nasopharyngeal epithelial cell lines (NP69 and NP460) were cultivated in Defined Keratinocyte-serum free medium (Invitrogen) [47, 48]. In total, 95 NPC and 13 normal nasopharyngeal specimens were obtained at the Department of Otolaryngology -Head & Neck Surgery, First Affiliated Hospital of Guangxi Medical University (Nanning, China), after written informed consent from donors, as previously described [6, 7]. They were anonymized for this study. Forty-seven cases were used for RNA extraction, another 40 were used for DNA extraction. Twelve normal controls provided enough biopsy material for both DNA and RNA extraction. A separate group of 69 NPC cases were included to evaluate the expression of UbcH8 in NPC tissues. Frozen sections were prepared from eight NPCs and one normal control. All were patients with primary NPC who underwent radiotherapy at the First Affiliated Hospital of Guangxi Medical University (Nanning, China) between 2005 and 2007. All the tissue samples were collected before radiotherapy. Tissue blocks were formalin-fixed and paraffin-embedded (FFPE). Patient demographics and clinical and follow-up information were retrieved retrospectively from medical records. Patient outcomes were assessed by interview, telephone and mail contacts from patients or relatives. Demographic data were collected, including patient age at diagnosis, sex, date of radiotherapy, date of last follow-up (if alive) and date and causes of death.

### Combined treatment with 5-aza-dC and TSA, and microarray analysis of global gene expression

Two NPC cell lines (CNE2 and HONE1) were used for microarray screening. A combined treatment with the demethylating agent 5-aza-2′-deoxycytidine (5-aza-dC) and the histone deacetylase inhibitor Trichostatin A (TSA), in NPC cell will synergize epigenetic upregulating of hypermethylated genes. The NPC cells were seeded at a density of 1 × 10^5^ per 100 mm dish and incubated 24 hrs, then treated with 10 μM 5-aza-dC (Sigma, St. Louis, Mo., USA) for 96 hrs, the medium containing 5-aza-dC was replaced every 24 hrs. Next, cells were treated with 100 nM TSA (Sigma) for another 24 hrs. The cells treated with DMSO were used for mock control. Total RNA from NPC cells was isolated using Trizol reagent (Invitrogen), and subjected to human cDNA microarray analyses by CapitalBio CO. (Beijing, China). Each sample was analyzed twice.

### Semi-quantitative reverse transcription-PCR (RT-PCR)

Total RNA from NPC cell lines, NPC primary tumor biopsies and NNE was isolated with TRIzol reagent (Invitrogen). First strand cDNA was synthesized with M-MLV reverse transcriptase (Promega, Madison, WI, USA) according to the manufacturer's instructions. Glyceraldehyde-3-phosphate dehydrogenase (*GAPDH*) was amplified from the same cDNA sample as the internal control. The amplified PCR products were then identified on 2% agarose gels. Images of ethidium bromide-stained agarose gels were acquired with a CCD camera (Bio-Rad Laboratories, Hercules, CA, USA) and semi-quantitative analysis was performed using the Quantity-one software, v4.4.0 (Bio-Rad).

### Immunohistochemistry

The Formalin-fixed and paraffin-embedded (FFPE) blocks from 69 NPC were cut into 3-μm-thick sections. The sections were deparaffinized in xylene, hydrated in graded alcohol solutions, and subjected to heat induced antigen retrieval in citrate buffer (0.01 M, pH 6.0) for 20 min at 96°C. Non-specific binding sites were blocked with serum-free blocking reagent (DAKO, Carpinteria, CA, USA) and anti-UbcH8 antibody (ab71800, Abcam, Cambridge, UK) was applied for 4 hrs at 25°C. Immunodetection was carried out with Envision reagent (anti-rabbit-HRP; DAKO) for 30 min. 3,3-Diaminobenzidine was then used as the visualizing substrate. Finally, sections were counterstained with hematoxylin. For a negative control, several NPC sections were incubated with isotype-matched IgG instead of the primary antibody.

Quantitative analysis was performed as described previously on an Olympus BH2 microscope with a computer-aided image-analysis system (QiuWei Inc., Shanghai, China), and images were captured using a digital camera (Nikon 4500, Tokyo, Japan) [49, 50]. The positive area and optical density of UbcH8-positive tumor cells were determined by assessment of three random microscopic fields (25 × 10) for each section. The immunohistochemical (IHC) index of the representative microscopic field = positive area × optical density (OD)/total area. Slides were blindly evaluated by two independent investigators who were unaware of the pathologic characteristics. The mean IHC indexes were used for the statistical analysis. For statistical analysis, the cases with IHC index lower than 0.285 were pooled into the low-expression group, and the cases with IHC index higher than 0.285 were pooled into the high-expression group.

### Methylation specific PCR (MSP)

The sodium bisulphite modification procedure was as described in previous study [7]. The methylation status of the *UBE2L6* promoter region was determined by MSP. Primers distinguishing unmethylated (U) and methylated (M) alleles were designed to amplify the sequence of *UBE2L6* from −141 to −26 bp relative to the transcription start point.

For each set of methylation specific PCR reactions, *in vitro*-methylated genomic DNA treated with sodium bisulphite served as a positive methylation control; a water blank control was also included. For cases with borderline results, PCR analyses were repeated.

### Bisulphite genomic sequencing

Sodium bisulphite-modified DNA was subjected to PCR with primers designed to amplify nucleotides from −204 to +156 bp relative to the transcription start point of the *UBE2L6* gene. PCR products were then gel purified and cloned using the pMD18-T Vector (Takara, Tokyo, Japan) and JM109 competent *E.coli* cells. Colonies were grown on agar plates, and 5 colonies of each sample were randomly selected. Plasmids were then isolated and purified. Sequencing was carried out using the BigDye terminator cycle sequencing kit 3.0 (Applied Biosystems, Foster City, CA, USA) on an ABI 3100 sequencer according to the manufacturer's guidelines. For all primer information in this study, see S1 table.

### Plasmid constructs, Western blot and immune-fluorescence analyses

The authenticity of Myc-VCP, Myc-UBE2L6 and HA-ISG15 was confirmed by DNA sequencing. The siRNA targeting UBE2L6 (sc-41685) and siRNA control (sc-37007) were purchased from Santa Cruz Biotechnology, Inc., CA, USA. Western blot and immune-fluorescence were done according to standard protocols. Antibodies and fluorescent dye used in this study are anti-*UBE2L6* (ab71800, Abcam), anti-Tubuline (T8203, Sigma), anti-Caspase 3 (8G10, Cell Signaling Technology, Beverly, MA, USA), anti-GAPDH (6C5, Santa Cruz), anti-Ki-67 (610969, BD Biosciences, San Diego, CA), anti-VCP (ab109240, Abcam), anti-ATGL (F7, Santa Cruz), anti HA (ab18181, Abcam). Secondary antibodies are anti-Rabbit/mouse IgG-HRP conjugate (Bio-Rad), Alexa fluor 594 goat anti-rabbit IgG (H+L), Alexa fluor 488 goat anti-mouse IgG (H+L) (Invitrogen). DAPI was from Beyotime (Hangzhou, China). IFN-αwas purchased from Life technologies (PHC4014, Grand Island, NY, USA).

The blots were visualized using enhanced chemiluminescent detection (Pierce, Rockford) with image detection on XDBF-1 film (Eastman Kodak, Rochester, NY, USA). Immuno-fluorescence images were taken on a Nikon A1 confocal microscope (Nikon, Tokyo, Japan).

### Human Cancer Pathway Finder PCR Array

The Human Cancer Pathway Finder Superarray (PAHS-033A; SABioscience Corp., Frederick, MD, USA) was used to explore transcriptional changes of 84 genes, which are representative of the six biological pathways involved in transformation and tumorigenesis, upon exogenous expression of *UBE2L6* in NPC cell line CNE2. All genes represented by the array showed a single peak on the melting curve characteristic to the specific products. Excel-based PCR Array Data Analysis Software provided by manufacturer (Qiagen, Valencia, CA, USA) was used for analysis of gene expression.

The genes found to be significantly modified by ectopic expression of *UBE2L6* were further confirmed by Semi-quantitative RT-PCR.

### Cell proliferation and colony formation assays

Cell proliferation was evaluated by trypan blue dye exclusion assay and immuno-staining with anti-Ki-67 antibody. An amount of 10^4^ cells of unmanipulated parental control cells, *UBE2L6* transfected and empty vector control cells were seeded in replicates into 6-well plates. From the following day on, cells were trypsinized and stained with a 0.4% trypan blue solution every 24 hrs. Dye negative cells were counted by use of a hemocytometer.

The protocol for colony formation has been described in the previous study [7].

### Annexin V-FITC/PI double-labeled flow cytometry

To determine apoptosis, the expression of annexin V-FITC and exclusion of propidium iodide (PI) (KeyGen BioTECH, Nanjing, China) were detected by double-labeled flow cytometry. Cells transfected with empty vector and *UBE2L6*-expressing vector were collected and washed with PBS 24 hrs after transfection, then resuspended in 100 μL binding buffer. Samples were incubated with 5 μL Annexin V-FITC in the dark for 10 min at 4°C, then the volume was adjusted to 500 μL with binding buffer. PI (5 μL) was added, and samples were incubated for another 10 min at 4°C. Fluorescence was measured with a flow cytometer (BD FACS Calibur, San Jose, CA, USA). The quantity of apoptotic cells was calculated as described [24, 51].

### Caspase-3 activity assay

The activity of caspase-3 was determined using a commercial Caspase-3 activity kit (Beyotime Institute of Biotechnology, Haimen, China) according to the manufacturer's instruction.

### Bodipy(403/503) staining and visualization

Bodipy (D3922, Molecular Probes, Carlsbad, Calif, USA) was diluted in DMSO at 1 μg/ml and applied on slides or tissue section for 30 min at RT. All samples were mounted in VECTASHIELD (Vector Laboratories, Burlingame, CA, USA) and covered with glass cover slips No. 1 (VWR).

We examined cell line samples under epifluorescent optics, and digital images were obtained with a laser scanning microscope (Leica Microsystem, Heidelberg, Germany), while frozen section with microscope from Olympus BX53 (Olympus, Tokyo, Japan).

### Statistical analysis

SPSS v11.5 (SPSS Inc., Chicago, IL, USA) was used for statistical analysis. The possible correlations between of methylated sample data and clinical pathological features were analyzed by Pearson chi-square test or Fisher's exact test.

The overall duration of survival was measured from the date of radiotherapy to the date of death from NPC. Deaths were the outcomes (events) of interest. Those patients who died from causes other than NPC, lost contact after last follow-up, or survived at the end of the study were considered to be censored. Survival curves were calculated using the Kaplan-Meier method in each group of patients with low UbcH8 staining and high UbcH8 staining, and differences were analyzed using the log-rank test.

## SUPPLEMENTARY FIGURES AND TABLES


