# Supplementary material for: Epigenetic downregulation of the ISG15–conjugating enzyme UbcH8 impairs lipolysis and correlates with poor prognosis in nasopharyngeal carcinoma
Source: Oncotarget. 2015 Oct 12;6(38):41077–91. doi: 10.18632/oncotarget.6218 (PMC4747391; doi:10.18632/oncotarget.6218)
Supplement: Supplementary file 1 [file oncotarget-06-41077-s001.pdf]

## SUPPLEMENTARY FIGURES AND TABLES

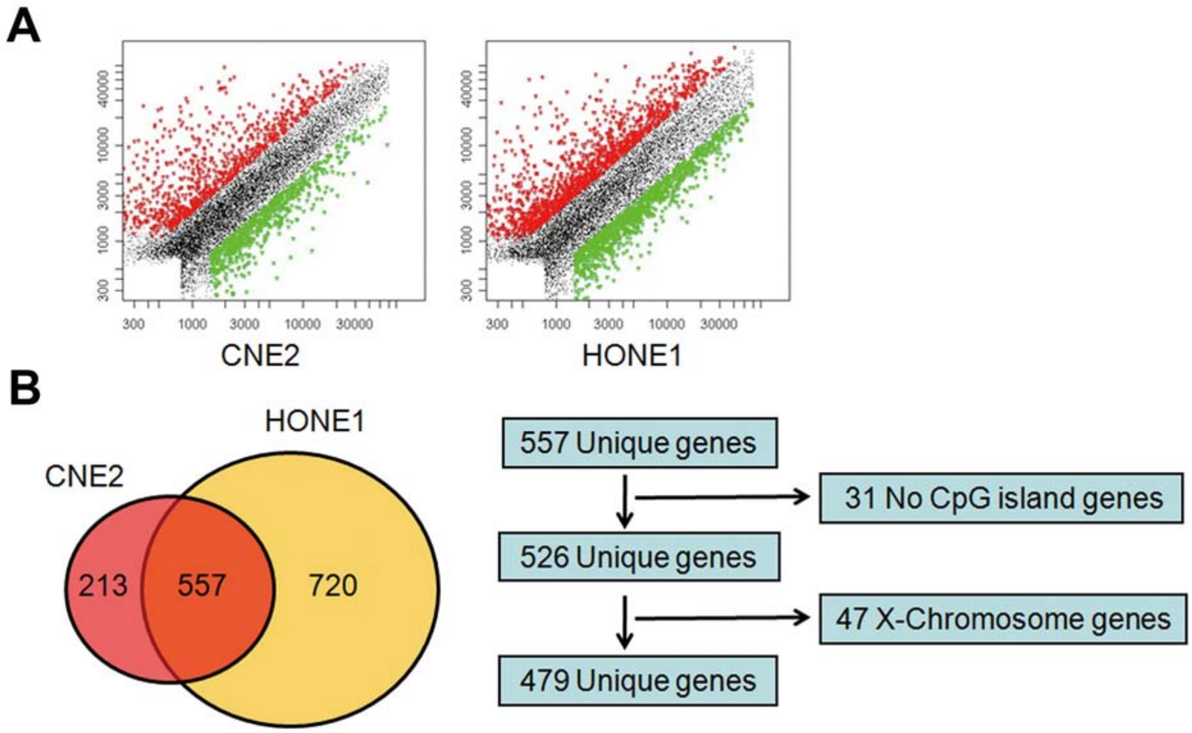


**Supplementary Figure S1: Genome-wide screening of genes inactivated by DNA methylation in multiple NPC cells. A.** Dot plots show the expression levels of genes altered by 5-aza-dC and TSA. Red dots represent genes, the expression levels of which were up-regulated after treatment, while green dots show genes that were down-regulated. **B.** Venn diagram depicts the distribution of up-regulated genes in two NPC cell lines. 557 genes were significantly up-regulated by 5-aza-dC and TSA in two NPC cell lines. Genes without CpG island in the 5′ region and genes located at the X chromosome were excluded, after which 479 candidates remained for further investigation.

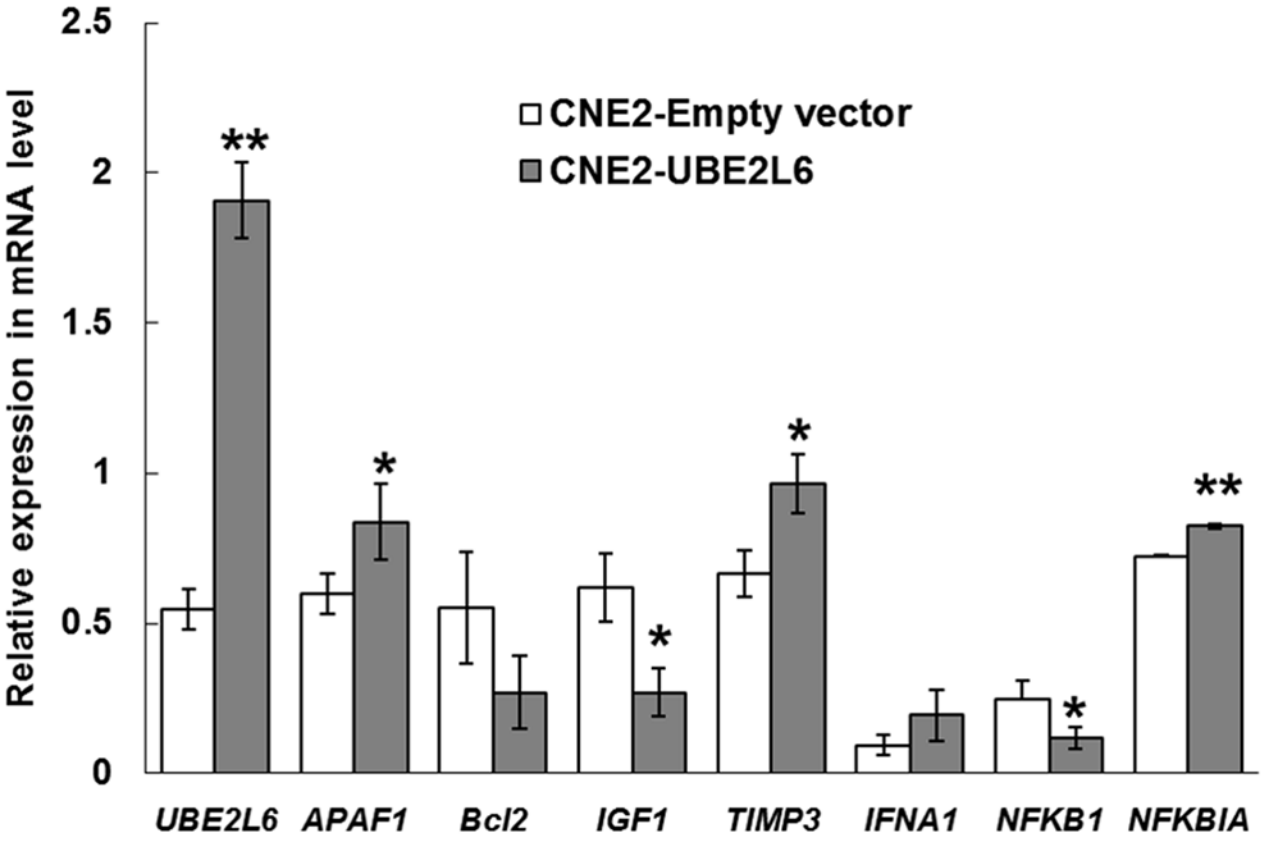


**Supplementary Figure S2: Gene expression analysis in CNE2 cells upon ectopic introduction of *UBE2L6* by semi-quantitative RT-PCR.** Relative expression levels obtained from 3 independent experiments are shown in bar graphs. There was a significant difference in expression of 7 genes, including apoptotic protease activating factor 1 (*APAF1*), B-cell CLL/lymphoma 2 (*Bcl-2*), insulin growth factor 1 (*IGF1*), human tissue inhibitor of metalloproteinases-3 (*TIMP3*), interferon alpha 1 (*IFNA1*), nuclear factor of kappa light polypeptide gene enhancer in B-cells inhibitor, alpha (*NFKBIA*) and nuclear factor of kappa light polypeptide gene enhancer in B-cells 1 (*NFKB1*).
$*p < 0.05$, $**p < 0.01$.

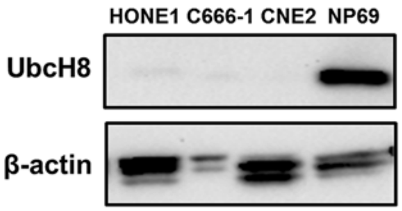


**Supplementary Figure S3: UbcH8 was downregulated in NPC cell lines.** Western blot analysis of the UbcH8 expression in NPC cell lines (HONE, C666–1 and CNE2) and normal epithelial cell line NP69. β-actin was used as an endogenous control.

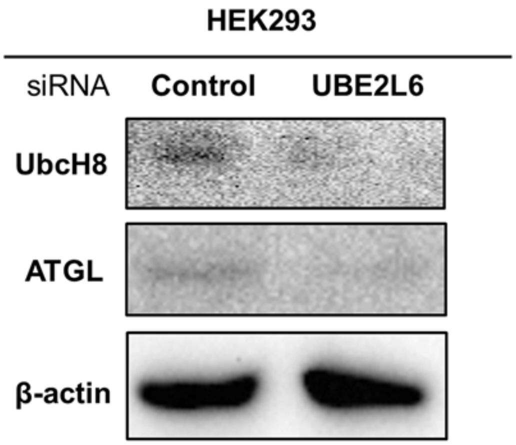


**Supplementary Figure S4: Knock-down of UbcH8 in HEK 293 cells downregulates the expression of ATGL.** Cells were transfected with either siRNA against UbcH8 (siUbcH8) or siRNA against irrelevant RNA (siControl). Forty-eight hours post-transfection cells were lysed and samples of total cell proteins were analysed by Western bolt, for UbcH8 and ATGL expression. β-Actin was used as an endogenous control.

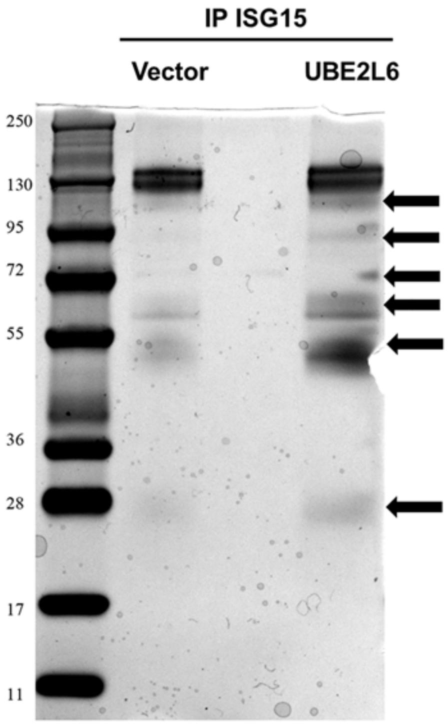


**Supplementary Figure S5: Exogenous expression of UbcH8 induces increased ISG15ylation.** CNE2 cells were transfected with either an empty vector or an *UBE2L6-coding* plasmid. Forty-eight hours post-transfection, cells were lysed and immunoprecipitation with ISG15 antibody were performed. ISG15ylated proteins were resolved on a polyacrylamide gel in denaturing condition and stained with silver. Arrows point to the ISG15ylated proteins.

**Supplementary Table S1: Primer sequences used in this study**

| | Primers | sequence(5′-3′) |
|---|---|---|
| RT-PCR | UBE2L6 | F:AAACACTGGCCGATCACCT |
| | | R:TGACCAAGATATCCTCCTCTGT |
| | APAF1 | F:CTGCCATAAGCCCTGTCCTCCA |
| | | R:ATTCTGGGTCCGGGTGCAGTT |
| | Bcl-2 | F:TGGCAGGAGGGGCAAGGTGGA |
| | | R:GCAGGTCGGTGAGCTGCCAGGATG |
| | IGF1 | F:CGGCAAAAGAAGTTGAACGAGTGG |
| | | R:TGCACAAGGGAGGTGTGTTGGTAA |
| | TIMP3 | F:GTAACCTGCGGATTGGCTTCG |
| | | R:GAAGCGGGTCACCTGGTCAGT |
| | IFNA1 | F:GTGGTGGGACCAGGGAGATTG |
| | | R:TTCAGCGTATCCGAGGACTTCTT |
| | NFKB1 | F:CCGGCTCGCAGGTCTCAAC |
| | | R:TCACCGGGCCGGAGGTTCAC |
| | NFKBIA | F:GCTCTCCTGTTGTGCTTCTCCACT |
| | | R:AGCTGCTTAATCTCCTCAGGGATG |
| | GAPDH | F:CTTCTGAGTTGCCCAGGAGACCACT |
| | | R:TCAACCACTCACACACACACAACCA |
| Real-time RT-PCR | ISG15 | F:CAACAAGCCCACAGGGTATGGCT |
| | | R:TGGGCATGTCGGTGTGGCGCT |
| | GAPDH | F:CAGGAGTTTGGGTCTGCAGTGTGA |
| | | R:TGGAGGAGGGAGTCCGATAGAAGC |
| Methylation Specific PCR | UBE2L6-M | F:GCATTGGCAAAGGTCGATTTGG |
| | | R:TCGCCGTGGACAGAGCAAGTT |
| | UBE2L6-U | F:CGAAGGTCCTACAGGGCCACAAC |
| | | R:CTCGCAAGAAATGCCCACATGAA |
| Bisulphite sequencing | UBE2L6-BISQ | F:AAGCTCACTGGCATGGCCTT |
| | | R:CTCTCTTCCTCTTGTGCTCTTG |
